# Costs of Hospitalization for Dementia in Urban China: Estimates from Two Urban Health Insurance Scheme Claims Data in Guangzhou City

**DOI:** 10.3390/ijerph16152781

**Published:** 2019-08-03

**Authors:** Hui Zhang, Donglan Zhang, Yujie Yin, Chao Zhang, Yixiang Huang

**Affiliations:** 1 School of Public Health, Sun Yat-sen University, No. 74, Zhongshan 2nd Road, Guangzhou 510080, China; 2 Department of Health Policy and Management, College of Public Health, University of Georgia, 100 Foster Road, Wright Hall 205D, Athens, GA 30602, USA; 3 Business School, Sun Yat-sen University, No. 135, Xinggang Xi Road, Guangzhou 510080, China

**Keywords:** dementia, health care costs, hospitalization, China, health insurance

## Abstract

*Background:* Dementia is one of the public health priorities in China. This study aimed to examine the hospitalization costs of patients with dementia and analyzed the factors associated with their inpatient costs. *Methods:* This was a prevalence-based, observational study using claims data derived from two urban insurance schemes during the period from 2008 through 2013 in Guangzhou. The extended estimating equations model was performed to identify the main drivers of total inpatient costs. *Results:* We identified 5747 dementia patients with an average age of 77.4. The average length of stay (LOS) was 24.2 days. The average hospitalization costs per inpatient was Chinese Yuan (CNY) 9169.0 (CNY 9169.0 = US$1479.8 in 2013). The mean inpatient costs for dementia patients with the Urban Employee-based Basic Medical Insurance (UEBMI) scheme (CNY 9425.0 = US$1521.1) were higher than those for patients with the Urban Resident-based Basic Medical Insurance scheme (CNY 7420.5 = US$1197.6) (*p* < 0.001). Having UEBMI coverage, dementia subtypes, having hypertension, being admitted in larger hospitals, and longer LOS were significantly associated with hospitalization costs of dementia. *Conclusions:* The costs of hospitalization for dementia were high and differed by types of insurance schemes. Dementia was associated with substantial hospitalization costs, mainly driven by insurance type and long LOS. These findings provided economic evidence for evaluating the burden of dementia in China.

## 1. Introduction

Due to the rapidly growing aging population, dementia is becoming a major public health issue worldwide [1,2]. A clinical diagnosis of dementia is based on a progressive cognitive decline and a clear departure from previous mental functioning [3]. A variety of pathopsychological progresses can lead to the clinical syndrome of dementia [3], for example, among them, the most common cause is Alzheimer’s disease (AD), followed by vascular dementia (VaD) [4]. A typical AD patient presents with other nerve system disorders such as behavioral or language deficits and visuospatial problems [3]. VaD is caused by insufficient blood supply to the brain following a series of minor strokes, which results in brain structural change and progressive cognitive decline [3,5]. A review of global epidemiology of dementia reported that the prevalence of all types of dementia was 6.4% in Europe (age-standardized), 14% in the United States (US) among people over 71 years old, 15.7% for illiterates and 7.16% for literates in Latin American countries [6]. The proportion of people with dementia living in developing countries was expected to rise from 60.1% in 2001 to 71.2% by 2040 [2]. The rapid growth of the elderly population results in an expected demographic shift in China as well. By 2010, there were 9.19 million people with AD and other subtypes of dementia in China and the prevalence of dementia was 2.6% at age of 65–69 and 60.5% at age of 95–99 [7]. The disease prevalence in China was forecasted to increase by more than 300% between 2001 and 2040 [7]. According to the 2015 World Alzheimer Report, China had the largest proportion (20%, 9.5 million) of dementia patients in 2015, and the number was predicted to exceed 16 million in 2030 [8].

With the growing number of patients, dementia imposes a significant financial burden on patients and their families, as well as on the health care systems. For example, in 2015, the estimated costs of dementia in the US was US$818 billion, equal to approximately 1% of the world gross domestic product (GDP) [9]. This figure had increased by 35% since 2010, and was estimated to reach around US$2 trillion in 2030 [9]. In China, the calculated total annual expenditure of dementia escalated from US$0.9 billion in 1990 to US$47.2 billion in 2010 and was predicted to reach US$69.0 billion by 2020 and US$114.2 billion by 2030 [10].

China expands health insurance coverage to the entire urban population with two social medical insurance programs—the Urban Employee-based Basic Medical Insurance scheme (UEBMI) and the Urban Resident-based Basic Medical Insurance scheme (URBMI) [11]. The UEBMI scheme covers urban employees working in cities, and the URBMI scheme covers urban residents who are unemployed, part-time workers or self-employed [12]. In urban regions, most dementia patients are covered by the UEBMI scheme or the URBMI scheme, but these two insurance schemes have different benefit designs and levels of financial protection [11]. The UEBMI scheme is financed by monthly payroll taxes from both employees and employers and provides the most comprehensive coverage; the URBMI scheme is financed by a fixed amount of money as premium contributions from the insured residents and local government but financial reimbursement remains limited [12]. Information about the direct medical costs associated with dementia is needed for insurance policy planning.

Many researchers have evaluated the direct medical costs of dementia in other countries [13,14,15,16,17]. In the US, the estimated direct medical costs attributable to dementia per person was US$8946 in 2010 [13]. In Germany, Schwarzkopf et al. [15] reported the yearly costs for inpatient stays per patient to be € 3914 in 2008. In Korea, the direct medical costs of dementia were US$4296 per patient in 2004 [16]. In Singapore, the direct medical costs of hospital admissions attributed to dementia among residents aged 60 years and above were US$ 7178 in 2013 [17]. In Europe, an observational study reported that the mean annual costs of inpatient care (excluding medication) were € 651 in 2006 for Northern Europe (Sweden, Denmark, UK, and Belgium), € 710 for Western Europe (Germany, Switzerland, the Netherlands and France) and € 450 for Southern Europe (Spain, Italy, Greece), respectively [18]. In China, few studies have analyzed the economic burden of dementia [10,19,20]. Jia et al. [20] investigated the socioeconomic costs of AD patients using data collected from 3098 patients in mainland China and found that the annual socioeconomic costs per patient were US$19,144.36 in 2015. Wang et al. [19] assessed the direct medical costs of patients with AD in Shanghai, China through interviews with 67 patients and their primary caregivers at the Department of Neurology in Ruijin Hospital. The study found that the mean direct medical costs were approximately US$707.7 in 2006 per year per patient. However, these two studies covered only AD patients rather than all dementia patients. The former study mainly focused on the macroeconomic burden of AD and the later study had a small sample size with only 67 patients recruited from one single hospital. In another study, Xu et al. [10] estimated the economic burden of dementia with data derived from the electronic health records of two medical facilities in Shandong province, China, and reported that the hospitalization costs were US$1004 in 2010. But the data source was from two hospitals. None of the abovementioned studies included the insurance information of dementia patients, nor did they analyze the drivers of the hospitalization costs.

Adding to the current literature, the present study aimed to examine the hospitalization costs of patients with dementia using the urban health insurance claims data and analyzed the factors associated with their inpatient costs in Guangzhou City, Southern China.

## 2. Materials and Methods

### 2.1. Data Source

Guangzhou city, the capital of Guangdong Province, is the largest and most developed city in Southern China. Thus, the study sample mainly represents the urban population in Southern China. We chose Guangzhou city in this study for two reasons. First, from a practical standpoint, we are only able to access insurance claims data from Guangzhou city while not from other cities or provinces due to administrative restrictions. Second, insurance policies in China vary between cities, and analyzing the per-person costs from insurance claims of an entire city minimizes potential selection issues caused by population or policy differences between cities. The data for this study were derived from the UEBMI and URBMI claims databases of Guangzhou city during the period from 2008 through 2013. This was the latest and de-identified claims dataset that was available for our research, due to administrative restrictions on access to the insurance claims data. The databases contained information on sociodemographic factors, direct medical costs of inpatient care based on actual payments to providers from a total of 309 medical institutions and explicit classification of medical conditions for a large sample of hospitalized patients receiving dementia treatment in Guangzhou city. The detailed benefits and reimbursement policies of the UEBMI and URBMI schemes were summarized in Table 1. The enrollees of these two urban schemes represented 96.6% of the registered residents in Guangzhou city [21].

### 2.2. Study Design

A prevalence-based and retrospective approach was applied to identify the total medical costs of inpatients with dementia. The study included all inpatient records of people admitted to hospitals in Guangzhou city with the primary diagnosis of dementia. We reviewed all the reimbursement claims submitted for inpatient care during 2008 and 2013 and subjects were selected using the International Classification of Diseases Tenth Edition (ICD-10), which included AD (F03 and G30), VaD (F01) and other types of dementia (G20) [16]. The last category included other unspecified dementia. In the meantime, we consulted with a neurologist to confirm the above disease subtypes. The study excluded patients under 18 years of age. A total number of 5747 dementia patients, including 5013 and 734 patients who were insured with the UEBMI scheme and the URBMI scheme, respectively, were finally selected.

### 2.3. Cost Estimation

The UEBMI and URBMI claims data provided information about direct medical costs of inpatients with different kinds of dementia from the healthcare system perspective, including the reimbursement amount paid by the health insurance scheme (UEBMI or URBMI) and out-of-pocket (OOP) spending paid by the patients. According to the classification of costs used in the UEBMI and URBMI schemes, the total inpatient medical costs were categorized as laboratory and diagnostic costs, non-medication treatment costs, medication costs, bed fees and the costs of other fees, including special caring fees and air-conditioning. Laboratory and diagnostic costs were the expenses of physical examinations and biochemical tests. Medication costs were separated into Traditional Chinese Medicine and western medicine spending. Non-medication treatment costs were the costs of any other treatments except for medication, which contained blood transfusion expenses, surgery fees, anesthesia charges, and costs for medical consumables. In this study all costs from 2008 to 2012 were adjusted to 2013 Chinese Yuan (CNY) value considering the urban resident’s Consumer Price Index (CPI) of Guangzhou city [21]. All costs were converted to US dollars. The annual exchange rate between US dollar and CNY in 2013 was: US$1 = CNY 6.196, based on the Bank of China data.

### 2.4. Measures and Variables

The dependent variable in this study was total hospitalization expenditures per inpatient. The primary independent variable was types of health insurance and was dichotomized as UEBMI and URBMI. Additional confounders included in the expenditure model were gender, age, disease subtype, comorbidities, hospital levels (primary, secondary, tertiary), length of stay (LOS), and years. Hospitals in China are categorized into three levels: primary (community health centers with only basic facilities and very limited inpatient capacity); secondary (hospitals with at least 100 inpatient beds providing acute care and preventive care services to at least 100,000 people); tertiary (major referral centers and teaching hospitals in provincial capitals and large cities) [22].

Predictors of total hospitalization costs for patients with dementia were identified based on the Andersen’s behavioural model [23]. Individual characteristics were chosen based on: (1) predisposing factors—existing conditions with predispose individuals to use or not use services (e.g., gender and age); (2) enabling factors—conditions that facilitate or impede the use of services (e.g., insurance type and hospital levels); and (3) need factors—conditions that healthcare providers recognize as requiring long-term medical treatment (e.g., disease subtype, comorbidities, LOS) [23].

Gender was dichotomized as male vs. female. Age was categorized into five groups: 18–50 years old, 50–59, 60–69, 70–79, 80–84, 85 and older. Disease type was grouped into three subtypes: AD, VaD, and other types of dementia. Comorbidities were measured as binary variables for the following conditions—whether having a diagnosis of hypertension, diabetes, coronary heart disease. Hospital level was classified into three levels: primary (level I), secondary (level II) and tertiary (level III). The LOS was grouped into five categories: less than 10 days, 11–15 days, 16–30 days, 31–60 days, longer than 60 days. Years were measured as binary variables for controlling the impact of policy changes across the years.

### 2.5. Statistical Analysis

Descriptive statistics (percentage, mean, median (25th–75th percentile) and standard deviation (SD)) were used for demographic information and costs. The Kruskal-Wallis test and Friedman’s two-way non-parametric ANOVA test were used to identify the differences in inpatient costs by types of insurance, because the value of medical spending often has a skewed distribution. In order to determine the main drivers of total inpatient costs and account for the skewness of cost data, in this study we applied the extension of generalized linear model (GLM)—the Extended Estimating Equations (EEE) model [24]. Advantage of the EEE model was that no retransformation was required because predictions were made on the raw cost scale [25]. In contrast to the difficulties of selecting the appropriate link function and distribution by the traditional GLM model, the EEE model allows for estimation of flexible link and variance functions using the data at hand, thereby reducing bias and inefficiency in estimation [24]. In order to deal with patients’ rehospitalization, we have corrected the standard errors for clustering at the patient level in the EEE model. We added interaction terms between insurance type and LOS categories in the regression analysis to explore possible interaction effects. All statistical calculations were performed using STATA version 12.0 (Stata Corporation, College Station, TX, USA).

### 2.6. Ethical Considerations

The present study was conducted according to ethical standards set by the institutional research committee. This research was approved by the Institutional Review Board of the School of Public Health, Sun Yat-sen University (Approval No. 2017012).

## 3. Results

### 3.1. Patient Characteristics

During the study period, a total of 5747 inpatients with dementia from the UEBMI and URBMI claims data in Guangzhou city were identified (see Table 2). Female accounted for a larger proportion of the overall sample (57.2%). The average age of the sample was 77.4 years old (SD = 10.0). Patients from the 70–80 age group (34.2%) outnumbered patients from the rest of the age groups. Among the total sample, 60% inpatients were AD subtype, 23.3% were VaD subtype, and 16.7% were other types of dementia. Overall 38.9% of the inpatients with dementia also suffered from hypertension, making it the major comorbidity among the enrolled inpatients. More than half of the inpatients (58.5%) received their treatment in tertiary hospitals, and 34.6% of the patients stayed in the hospitals for 15–30 days. The mean LOS was 24.2 days. Most of the patients in our study were covered by the UEBMI scheme (87.2%) (Table 2).

### 3.2. Hospitalization Costs of Dementia and Cost Composition by Types of Insurance

Overall, the mean total inpatient costs of patients with dementia was CNY 9169.0 (CNY 9169.0 = US$1479.8, in 2013) (see Table 3)., Non-medication treatment costs and medication costs were two of the biggest contributors to the total expenditures, accounting for 38.5% and 38.4% respectively. Laboratory and diagnostic costs ranked the third in all cost drivers (9.7%), followed by the bed fees (9.2%).

When comparing the hospitalization costs by insurance status, the mean total inpatient costs for patients with dementia under the UEBMI scheme (CNY 9425.0 = US$1521.1) was higher than the patients under the URBMI scheme (CNY 7420.5 = US$1197.6) (*p* < 0.001). However, the percentage OOP expenses of the total costs for the URBMI scheme patients (30.0%) was nearly 2 times that for the UEBMI scheme patients (18.1%), indicating the underlying differences in benefit packages of the two insurance schemes. Regarding cost composition, the biggest cost component in the UEBMI group was non-medication treatment costs (38.7%), while the biggest cost component in the URBMI group was medication costs (37.9%).

### 3.3. Patient Characteristics Associated with Inpatient Costs by Types of Insurance

Inpatient costs between the UEBMI subgroup and URBMI subgroup significantly differed according to age groups, dementia subtypes, comorbidities, hospital levels, and LOS (*p* < 0.01) (see Table 4). Among all age groups for the entire sample, patients aged 50–60 had the highest mean inpatient costs (CNY 10,278.0 = US$1658.8), whereas patients aged over 85 had the lowest costs. This result was found for both the UEBMI and URBMI subgroups. Overall, the mean inpatient costs for patients with VaD (CNY 9934.0 = US$1603.3) were higher than that for patients with AD (CNY 8251.9 = US$1331.8). It was worth mentioning that the longest LOS (>60 days) incurred the highest mean medical costs for both the UEBMI and URBMI subgroups. The highest mean inpatients costs were found in patients staying in tertiary hospitals, nearly two times as high as the costs among patients being hospitalized in secondary hospitals for the overall sample.

### 3.4. Influential Factors of Total Inpatient Costs

This study found that insurance type, dementia subtypes, comorbidities, hospital levels and LOS were significantly associated with inpatient costs of dementia (*p* < 0.01) for the overall sample (see Table 5). Compared with patients under the URBMI scheme, the inpatient costs of dementia for the UEBMI beneficiaries were CNY 1714.2 (US$276.7) significantly higher, after controlling for the cofounders (*p* < 0.01).

When further adding the interaction terms in the model, there were statistically significant interactions between insurance type (UEBMI) and LOS (30 < Days ≤ 60; >60 Days) categories (*p* for interaction <0.01) (see Table 6). The model indicated that compared with patients under the URBMI scheme, the UEBMI enrollees had significantly higher hospitalization costs of dementia when the LOS was longer than 30 days.

Different findings were observed between the UEBMI subgroup and URBMI subgroup (see Table 5). Gender was a significant factor only among the UEBMI subgroup, and male patients under the UEBMI scheme had significantly higher hospitalization costs than their female counterparts (CNY 594.1; *p* < 0.05). Among the UEBMI subgroup, the hospitalization costs for VaD inpatients and patients with other types of dementia were CNY 1317.3 (US$212.6) and CNY 1567.3 (US$253.0) significantly higher respectively compared to the AD inpatients (*p* < 0.01). Patients with hypertension among the UEBMI subgroup incurred significantly higher inpatient costs (CNY 642.7 = US$103.7) (*p* < 0.01), while patients with diabetes among the URBMI subgroup had significantly higher hospitalization expenses (CNY 3724.5 = US$601.1) (*p* < 0.05). Dementia patients stayed at tertiary hospitals had CNY 6153.0 (US$993.1) higher medical expenditures among the UEBMI subgroup and CNY 5097.8 (US$822.8) higher among the URBMI subgroup, compared with patients staying at primary hospitals (*p* < 0.01). Among the UEBMI and URBMI subgroups, patients with longer LOS incurred significantly higher inpatient costs after adjusting for other factors. Compared with LOS less than 10 days, hospitalization costs for the longest LOS group (>60 days) was CNY 16,933.1 (US$2732.9) higher among the UEBMI patients and CNY 7500.0 (US$1210.5) higher among the URBMI patients (*p* < 0.01).

## 4. Discussion

It was strategically important to understand the hospitalization costs of patients with dementia and the key drivers of the medical expenses in China due to the high burden of the disease. The present study was an observational study conducted with a large dementia sample in Guangzhou city. We found that the mean total direct inpatient costs of dementia were CNY 9169 (US$1479.8). The non-medication treatment costs and medication costs were the biggest contributors to the total expenses. The type of insurance schemes (UEBMI), different dementia subtypes, comorbidities (having hypertension), being admitted in the secondary and tertiary hospitals, and longer LOS were significantly associated with total inpatient costs of dementia patients. When further exploring the interaction effect between insurance type and LOS, we found that the UEBMI enrolees had significantly higher hospitalization costs as compared to the URBMI enrolees when the LOS exceeded 30 days. This was the first study using data from two urban health insurance claims data of an entire city to investigate the healthcare costs for hospitalization of dementia and compare the costs under two different urban insurance schemes as well as to identify the key drivers of inpatient costs for dementia in China.

Comparing our findings to studies in other developed countries, a significant wide gap was observed in the direct medical costs incurred in dementia patients. To compare the costs of different countries in different study period, we derived 2013 US dollar value by using consumer price indices of study countries in the years of costs and purchasing power parity (PPP) exchange rate in 2013 from the Organization for Economic Co-operation and Development (OECD) [26]. The average hospitalization cost found in this study (US$1479.8) was much lower than the costs in U.S. [13], Germany [15], Korea [16], and Singapore [17], but it was slightly higher than those inpatient care in some European countries [18]. The detailed comparison of hospitalization costs for dementia patients by country was summarized in Table 7. However, the international comparison of the healthcare cost for dementia was limited by differences in the calculation methods (prevalence or incidence-based), different categories of costs included (inpatient, outpatients, nursing home or formal home care services) and different cognitive impairment stages of dementia (mild, moderate or severe) included in those studies. The variation also lied mainly in different health care systems across the countries.

Regarding the cost composition, the non-medication treatment costs (38.5%) and medication costs (38.4%) took up the biggest proportion of the inpatient costs for dementia patients in this study. The non-medication treatment costs for dementia patients included cognitive behavior therapy [27], bright-light therapy [28], art therapy [29] and reminiscence therapy [30] during hospitalization. Acetylcholinesterase inhibitors and memantine, an N-methyl-D-aspartic acid receptor antagonist were medications currently licensed for the treatment of dementia [3]. In northern, western and southern Europe [18], the medical costs for non-medication treatment and medication were 53.7% and 46.2%, 49.6% and 50.3%, 49.6% and 50.3% respectively. Generally, it was difficult to compare the international studies on the composition of direct medical costs of dementia due to the significant differences in study methods, the subtypes of dementia included and the cost components considered [14]. When comparing cost components of dementia with previous studies in China, Zhou and Zhen [31] reported a consistent result: the biggest contributor to the direct medical costs was non-medication treatment and medication, taking up 29.78% and 31.8% of the total costs, respectively. The hospitalization costs of the present study included medication and non-medication treatment costs, while the inpatient costs in previous European study [18] mentioned above did not cover medication costs but mainly focused on inpatient care, which may explain the relatively higher hospitalization costs in our study.

When comparing the average costs of dementia reported in this study with previous China-based studies, our finding was higher. Wang et al. [19] reported the average direct medical care costs of AD were CNY 5640 (US$707.7, in 2006) per patient, while Xu et al. [10] estimated costs of dementia to be US$1004 in 2010 per case. It was inappropriate to compare the average direct medical costs of dementia per patient reported in this study to the other two China-based studies [10,19], since they narrowly focused on only one subtype of dementia, namely AD. Our samples included more subtypes of dementia than previous studies. The VaD subtype was more likely to incur higher medical expenditures than the AD subtype, which might explain the higher inpatient costs found in the present study.

This study also examined the differences in hospitalization costs of dementia between two urban health insurance schemes for the first time while the previous China-based studies did not mention. The patients enrolled in the UEBMI scheme incurred higher inpatient costs than the URBMI patients, but the UEBMI patients had a lower percentage of OOP expenses. This finding was consistent with previous research showing how different types of insurance affected health care utilization and costs among patients with other diseases in China [32,33,34]. The type of health insurance schemes was also found to be a significant predictor of the hospitalization costs of dementia, and the inpatient costs of dementia was significantly higher for patients with the UEBMI scheme. There are some reasons for this finding. First, since the UEBMI and URBMI schemes covered specific groups (see Table 1) with different financing mechanisms and reimbursement policies, the inequality in health care utilization and medical costs may exist among patients enrolled in different types of health insurance schemes [34]. Thus, the inpatients of dementia under UEBMI scheme, who have higher income and higher reimbursement ratios for more comprehensive services may seek better medical treatment at higher-level institutions and be more likely to spend more on health care. Second, the URBMI scheme does not provide adequate financial protection to cover the health care services for beneficiaries, thus may discourage the URBMI patients to use expensive services [32]. The health and economics outcomes may be worse among patients enrolled in the URBMI in which the scale of financing was insufficient [35]. As a result, inpatients who were insured by the URBMI scheme have worse personal financial status and relatively limited health care access, therefore, they would have lower health care expenses. Therefore, reducing the gap in reimbursement rates among these two insurance schemes should be a focus of insurance policy planning in China, as was shown in our results, dementia patients covered by the URBMI scheme had a higher percentage of OOP expenses than UEBMI patients. In order to narrow the disparities between these two different insurance schemes in financing, budget and benefit packages, we propose that the UEBMI and URBMI schemes should be further consolidated to be an integrated health insurance program in China.

In the present study, we found that compared with AD patients, the inpatient costs for VaD patients were significantly higher. Societies where hypertension was the major problem seemed to have a proportionally high prevalence of VaD [6]. More variable cognitive changes in VaD than in AD made it harder to be diagnosed: standard screening tests devised to detect AD proved less sensitive for VaD [4]. Thus, in order to pick up deficits in VaD patients, more tests such as the Montreal cognitive assessment scale were needed [4], leading to an increase in the total hospitalization costs. Mortality was also found higher in VaD [4], indicating a more serious condition in VaD patients that resulted in higher hospital utilizations than AD patients. In line with this study, Fillit and Hill [36] also reported the same difference between costs for patients with VaD and AD, where hospital costs and hospital days were two times greater for VaD patients. Due to the nature of symptoms of VaD, more complex medical treatments resulted in excess medical expenditures and extended hospital stays.

Consistent with the previous literature [37], hypertension was found positively related to higher hospitalization expenditures among dementia patients. Compared with dementia patients without comorbidity, the existence of hypertension among dementia patients increased the severity of their conditions, inducing more medical resources consumption [37]. Consistent with other studies in China [38,39,40], patients hospitalized at higher level institutions had higher inpatients costs. Patients admitted to higher level hospitals may often in more acute and sever conditions and thus need more expensive consumables and advanced diagnostic instruments which were not equipped in primary hospitals [39]. High-level hospitals had better resource distribution and patients in higher level hospitals would have higher medical services demand and utilization leading to higher medical costs in China [38].

We found that longer LOS was related to higher medical expenditures, which was consistent with previous studies [36]. After examining the interaction effects, we found that LOS differed by insurance type. The UEBMI scheme patients had significantly higher hospitalization costs when the LOS exceeded 30 days, which suggested that strategies to reduce UEBMI patients’ LOS may be needed to reduce the inpatient costs of dementia. The mean LOS per case was 24.2 days in this study, much longer than that in Australia (16.4 days) [41], Romania (5.49 days) [18] and Ireland (23.7 days) [42]. The progressive and unpredictable nature of dementia posed enormous challenges to caregivers. Long survival time, fluctuating functional level and serious safety issue caused by psychopathologic behaviours such as wandering and hoarding contributed to the need of caregivers’ full-time care and specialized caring skills [43]. Owning to the one-child policy, a named 4-2-1 family structure (e.g., a family constituted by four grandparents, two parents and one child) has become the mainstream family structure in China [44]. In the coming decades, the number of old people will keep rising as the number of available family caregivers shrinks [1]. Considering this socioeconomic transition, the traditional family-dependent long-term care (LTC) of the past would no longer suffice [45]. Comparing to the community-based dementia care setting which provided patients with well-established long-term care and rehabilitation system [15], and well-established day centres and geriatric residences [46] in Western countries, the domestic LTC system in China is in its early stages: both institutional and community-based services barely exist at the time and could hardly meet the growing need for LTC in China [47]. Currently, tentative LTC reforms have been made by local governments, but public financial support is only available for partial nursing institutions and community health services leaving the main burden of expenses to be borne by individuals and families [48]. Though public funding constitutes the major source of LTC financing in China, a large proportion of costs is still paid by service users themselves [49]. Given the lack of post-dementia rehabilitation centres and financial support for long-term care of dementia, families would rather send patients with dementia to hospitals where health insurance is the primary payer for their healthcare expenditures, resulting in lengthy hospital stays and a huge waste of scarce medical resources. Hence, there is an increasing need for the Chinese government to support the establishment of an LTC insurance system that allocates LTC expenses more fairly across the government, individuals, and families, as population aging will double the LTC expenses by 2030 [48].

There were some limitations in this study. First, this study only examined hospitalization costs. The costs of outpatient care, indirect costs due to loss of productivity and family members’ informal care were not analysed since our dataset did not include this information. Thus, we may underestimate the total medical expenditures of dementia in China. Second, the study population was limited to urban insurance beneficiaries in one city of China and did not include rural patients, from which the sample cannot represent the whole Chinese population. Third, disease severity, which was often measured by the Mini-Mental State Examination and an important predictor of costs, was not included in the analysis because such data were not available in the claims data.

## 5. Conclusions

The costs of hospitalization for dementia were high and differed by types of insurance schemes in China. Dementia was associated with substantial hospitalization costs, mainly driven by the insurance type and lengthy hospital stays. The findings of this study could provide valuable information for understanding the burden of dementia and evaluating the health insurance policy in China. Future studies could consider including outpatient costs, direct non-medical costs, and indirect costs to measure the societal costs of dementia for a better understanding of the economic burden of dementia in China. In addition, studies could also consider analysing dementia patients covered by both urban and rural insurance schemes, as well as including patients from other cities in China, and collecting information on disease severity in future analysis.

The findings of this study have important policy implications for reducing the costs of hospitalization for dementia patients and improving the health insurance system in China. First, given the differences in reimbursement rates and benefit packages between the UEBMI and URBMI schemes, we suggest that these two urban health insurance schemes should eventually be consolidated to be an integrated insurance program in China. Second, efforts to reduce hospital LOS such as establishing more community-based care facilities, day care centres and promoting home-based care could be viable measures to contain hospitalization costs of dementia in China. Third, besides the basic health insurance schemes, China should establish a long-term care insurance program to support dementia patients who receive long-term institutional or residential care, which could reduce the economic burden of China’s basic health insurance funds through reducing the lengthy hospital LOS.

## Figures and Tables

**Table 1:** Comparison of UEBMI and URBMI policies for dementia patients in Guangzhou city in 2013.

Inception Year	UEBMI			URBMI		
	2002			2008		
Eligible population	Urban employed (Employees; Retirees)			Urban non-employed (Children & full-time Students; Unemployed adults; Elderly residents not covered by the UEBMI scheme)		
Sources of funding	The employers contribute 6% of the employee’s salary whilst the employees contribute 2%; Retirees are exempted from premium contribution			Government subsidy (70%) and individual premium (30%) CNY440 to CNY1800 per person per year for residents (including government subsidy)		
Accounts	Medical Savings Account (including employee contributions and 30% of employer contributions) for outpatient care; Social Risk-pooling Account (70% of employer contributions) for inpatient care and critical (i.e., chronic or fatal diseases) outpatient care			Social Risk-pooling Account (all funds) for inpatient care and critical (i.e., chronic or fatal diseases) outpatient care		
	Inpatient			Inpatient		
Deductible: (Inpatient care)	Employees	Primary hospitals	CNY 400	Children & students	Primary hospitals	CNY 120
		Secondary hospitals	CNY 800		Secondary hospitals	CNY 240
		Tertiary hospitals	CNY 1600		Tertiary hospitals	CNY 480
	Retirees	Primary hospitals	CNY 280	Unemployed adults and Elderly residents	Primary hospitals	CNY 280
		Secondary hospitals	CNY 560		Secondary hospitals	CNY 560
		Tertiary hospitals	CNY 1120		Tertiary hospitals	CNY 1120
Reimbursement rate * (Inpatient care)	Employees	Primary hospitals	90%	Children & students	Primary hospitals	85%
		Secondary hospitals	85%		Secondary hospitals	75%
		Tertiary hospitals	80%		Tertiary hospitals	65%
	Retirees	Primary hospitals	93%	Unemployed adults and Elderly residents	Primary hospitals	75%
		Secondary hospitals	89.5%		Secondary hospitals	65%
		Tertiary hospitals	86%		Tertiary hospitals	55%
Reimbursed ceiling	Six times of local employees’ annual average wage			Six times of local household disposable income		
(Inpatient care)	CNY 382,512			CNY 228,324		

Notes: Policy information was obtained from Statistical Bulletin of Guangzhou Social Insurance Bureau, and policy documents. * The percentages were the reimbursement rates of the eligible medical expenses that could be reimbursed from the Social Risk-pooling Account in Guangzhou. Abbreviations: UEBMI, Urban Employee-based Basic Medical Insurance scheme; URBMI, Urban Resident-based Basic Medical Insurance scheme; CNY: Chinese Yuan.

**Table 2:** Dementia patient characteristics.

Patient Characteristics	Overall	UEBMI	URBMI
No. Patients	5747	5013	734
Gender			
Female	3288.0 (57.2)	2893.0 (57.7)	395.0 (53.8)
Male	2459.0 (42.8)	2120.0 (42.3)	339.0 (46.2)
Age (years)			
Mean ± SD	77.4 ± 10.0	77.6 ± 9.7	76.1 ± 12.0
Median (25th–75th)	79.0 (72.0–84.0)	79.0 (73.0–84.0)	76.0 (70.0–85.0)
Age group			
18 ≤ age < 50	56.0 (1.0)	30.0 (0.6)	26.0 (3.5)
50 ≤ age < 60	332.0 (5.8)	280.0 (5.6)	52.0 (7.1)
60 ≤ age < 70	670.0 (11.7)	582.0 (11.6)	88.0 (12.0)
70 ≤ age < 80	1967.0 (34.2)	1711.0 (34.1)	256.0 (34.9)
80 ≤ age < 85	1391.0 (24.2)	1267.0 (25.3)	124.0 (16.9)
≥85	1331.0 (23.2)	1143.0 (22.8)	188.0 (25.6)
Insurance type			
UEBMI	5013.0 (87.2)	5013.0 (100.0)	0.0 (0.0)
URBMI	734.0 (12.8)	0.0 (0.0)	734.0 (100.0)
Disease type			
AD	3448.0 (60.0)	2911.0 (58.1)	537.0 (73.2)
VaD	1338.0 (23.3)	1214.0 (24.2)	124.0 (16.9)
Others	961.0 (16.7)	888.0 (17.7)	73.0 (9.9)
Comorbidities			
None	3258.0 (56.7)	2698.0 (53.8)	560.0 (76.3)
Hypertension	2238.0 (38.9)	2067.0 (41.2)	171.0 (23.3)
Diabetes	413.0 (7.2)	407.0 (8.1)	6.0 (0.8)
Coronary	779.0 (13.6)	742.0 (14.8)	37.0 (5.0)
Hospital level			
Primary	358.0 (6.2)	330.0 (6.6)	28.0 (3.8)
Secondary	2026.0 (35.3)	1803.0 (36.0)	223.0 (30.4)
Tertiary	3363.0 (58.5)	2880.0 (57.5)	483.0 (65.8)
Length of stay (days)			
Mean ± SD	24.2 ± 21.6	22.8 ± 19.9	33.7 ± 29.0
Median (25th–75th)	17.0 (12.0–30.0)	17.0 (12.0–29.0)	29.0 (11.0–34.0)
Days ≤ 10	1137.0 (19.8)	972.0 (19.4)	165.0 (22.5)
10 < Days ≤ 15	1314.0 (22.9)	1209.0 (24.1)	105.0 (14.3)
15 < Days ≤ 30	1990.0 (34.6)	1823.0 (36.4)	167.0 (22.8)
30 < Days ≤ 60	830.0 (14.4)	685.0 (13.7)	145.0 (19.8)
>60 Days	476.0 (8.3)	324.0 (6.5)	152.0 (20.7)

*n* (%) for categorical variables and mean ± standard deviation (SD) or median (25th–75th) for continuous variables; UEBMI, Urban Employee-based Basic Medical Insurance scheme; URBMI, Urban Resident-based Basic Medical Insurance scheme; AD, Alzheimer’s disease; VaD, Vascular dementia; Others, other forms of dementia.

**Table 3:** Direct inpatient costs by types of insurance.

Direct Inpatient Costs	Overall	UEBMI	URBMI	*p*-Value
No. Patients	5747	5013	734	
Total inpatient costs				0.000
Mean (CNY)	9169.0	9425.0	7420.5	
SD	7899.1	8239.6	4625.2	
Laboratory and diagnostic costs				0.000
Percentage of total inpatient cost (%)	9.7	10.1	6.1	
Mean (CNY)	887.7	951.0	455.3	
SD	1599.2	1662.3	969.8	
Non-medication treatment costs				0.000
Percentage of total inpatient cost (%)	38.5	38.7	36.4	
Mean (CNY)	3526.6	3647.5	2700.8	
SD	3603.9	3727.6	2454.2	
Medication costs				0.000
Percentage of total inpatient cost (%)	38.4	38.5	37.9	
Mean (CNY)	3524.3	3628.2	2814.4	
SD	3946.1	4116.6	2370.0	
Bed fees				0.000
Percentage of total inpatient cost (%)	9.2	8.7	13.9	
Mean (CNY)	847.2	820.5	1029.0	
SD	779.2	752.7	920.5	
Other fees				0.000
Percentage of total inpatient cost (%)	4.2	4.0	5.7	
Mean (CNY)	383.3	377.8	421.0	
SD	414.9	425.2	334.6	
Out-of-pocket spending				0.539
Percentage of total inpatient cost (%)	19.3	18.1	30.0	
Mean (CNY)	1771.9	1705.9	2222.3	
SD	1841.8	1683.0	2643.9	

*p*-values are based on the Kruskal-Wallis test. All costs were based on a constant 2013 Chinese Yuan (CNY); UEBMI, Urban Employee-based Basic Medical Insurance scheme; URBMI, Urban Resident-based Basic Medical Insurance scheme; CNY: Chinese Yuan; SD, Standard deviation.

**Table 4:** Dementia patient characteristics associated with inpatient costs.

Patient Characteristics	Overall		UEBMI		URBMI		*p*-Value
No. Patients	*n* = 5747		*n* = 5013		*n* = 734		
	Mean	SD	Mean	SD	Mean	SD	
Gender							0.077
Female	8880.5	7905.2	9143.9	8239.4	6951.2	4336.8	
Male	9554.8	7875.9	9808.6	8226.3	7967.3	4889.8	
Age group							0.000
18 ≤ age < 50	9310.4	3822.0	10,122.4	4244.6	8373.4	3087.5	
50 ≤ age < 60	10,278.0	8611.8	10,523.7	9190.0	8954.9	4146.0	
60 ≤ age < 70	9721.0	7869.2	10,024.8	8119.6	7712.1	5586.3	
70 ≤ age < 80	9936.5	8828.9	10,351.7	9282.8	7161.5	3772.2	
80 ≤ age < 85	8585.7	7212.7	8709.8	7438.3	7317.4	4081.1	
≥85	8083.9	6848.0	8237.7	7014.6	7148.5	5658.5	
Insurance type							\
UEBMI	9425.0	8239.6	9425.0	8239.6	\	\	
URBMI	7420.5	4625.1	\	\	7420.5	4625.1	
Disease type							0.000
AD	8251.9	7394.7	8477.4	7820.9	7029.8	4219.8	
VaD	9934.0	7451.2	10,188.6	7670.2	7442.2	4052.7	
Others	11,394.2	9539.4	11,487.7	9720.5	10,257.8	6918.4	
Comorbidities							0.000
None	8808.6	7965.6	9065.8	8452.3	7569.3	4811.2	
Hypertension	9730.3	8058.1	9971.0	8266.4	6821.4	3842.4	
Diabetes	10,118.2	7378.7	10,077.9	7407.1	12,847.0	4703.9	
Coronary	10,019.0	7497.4	10,116.1	7619.1	8071.7	3979.4	
Hospital level							0.000
Primary	5538.6	3256.6	5615.4	3264.1	4633.1	3079.7	
Secondary	6733.0	5427.8	6879.0	5642.8	5552.9	2950.5	
Tertiary	11,023.0	8907.9	11,455.4	9337.9	8444.4	4987.5	
Length of stay (days)							0.000
Days ≤ 10	6873.5	3583.1	7044.4	3566.8	5866.5	3523.0	
10 < Days ≤ 15	8996.5	4543.6	9132.3	4575.5	7432.1	3844.0	
15 < Days ≤ 30	9056.7	6012.4	9173.4	5878.5	7783.5	7215.9	
30 < Days ≤ 60	8504.1	8758.4	8933.3	9496.0	6476.4	2876.2	
>60 Days	16,757.1	17,622.2	20,114.1	20,449.4	9601.3	2574.5	

*p*-values are based on the Friedman’s two-way non-parametric ANOVA test. All costs were based on a constant 2013 Chinese Yuan (CNY); UEBMI, Urban Employee-based Basic Medical Insurance scheme; URBMI, Urban Resident-based Basic Medical Insurance scheme; SD, Standard deviation; AD, Alzheimer’s disease; VaD, Vascular dementia; Others, other forms of dementia.

**Table 5:** Factors associated with total inpatient costs (EEE Model).

Influential Factors	Overall			UEBMI			URBMI		
	*n* = 5747			*n* = 5013			*n* = 734		
	Coef.	Adjusted Std. err.	Marginal Effect	Coef.	Adjusted Std. err.	Marginal Effect	Coef.	Adjusted Std. err.	Marginal Effect
Gender									
Female (Reference)									
Male	0.043	0.028	409.2	0.063 **	0.024	594.1	0.033	0.089	262.3
Age group									
18 ≤ age < 50 (Reference)									
50 ≤ age < 60	0.055	0.116	540.0	−0.053	0.108	−494.9	0.240	0.168	2342.6
60 ≤ age < 70	0.073	0.097	720.2	−0.020	0.083	−186.9	0.233	0.217	2184.3
70 ≤ age < 80	0.092	0.093	886.8	−0.016	0.077	−152.5	0.160	0.188	1352.3
80 ≤ age < 85	0.043	0.096	415.9	−0.060	0.079	−562.1	0.260	0.211	2393.7
≥85	0.074	0.095	722.7	−0.057	0.078	−530.1	0.352	0.208	3645.1
Insurance type									
URBMI (Reference)									
UEBMI	0.200 ***	0.046	1714.2	\	\	\	\	\	\
Disease type									
AD (Reference)									
VaD	0.136 ***	0.030	1354.4	0.135 ***	0.027	1317.3	0.069	0.089	564.4
Others	0.212 ***	0.033	2209.9	0.158 ***	0.03	1567.3	0.426 ***	0.12	3982.2
Comorbidities									
None (Reference)									
Hypertension	0.091 ***	0.028	870.3	0.067 ***	0.026	642.7	0.101	0.083	793.2
Diabetes	0.049	0.037	481.2	0.040	0.036	384.7	0.370 **	0.166	3724.5
Coronary	0.049	0.033	471.9	0.046	0.03	445.7	0.082	0.103	672.1
Hospital level									
Primary (Reference)									
Secondary	0.252 ***	0.039	2702.0	0.215 ***	0.032	2128.5	0.213	0.197	1950.9
Tertiary	0.721 ***	0.054	6129.6	0.693 ***	0.048	6153.0	0.731 ***	0.23	5097.8
Length of stay (days)									
Days ≤ 10 (Reference)									
10 < Days ≤ 15	0.301 ***	0.024	3297.5	0.308 ***	0.023	3168.3	0.216 ***	0.077	1908.8
15 < Days ≤ 30	0.355 ***	0.029	3822.4	0.372 ***	0.028	3753.4	0.218 **	0.105	1869.4
30 < Days ≤ 60	0.253 ***	0.050	2778.1	0.295 ***	0.053	3073.4	−0.030	0.112	−228.5
>60 Days	0.934 ***	0.092	16,731.8	1.178 ***	0.141	16,933.1	0.532 ***	0.138	7500.0
Year									
year 2008 (Reference)									
year 2009	0.169 ***	0.047	1746.7	0.178 ***	0.039	1782.0	0.146 ***	0.053	1280.9
year 2010	0.181 ***	0.048	1905.5	0.149 ***	0.035	1488.9	0.134	0.120	1176.5
year 2011	0.135 ***	0.042	1357.7	0.109 ***	0.032	1063.4	0.290 ***	0.109	2813.6
year 2012	0.174 ***	0.042	1755.7	0.124 ***	0.032	1199.2	0.328 ***	0.109	3002.9
year 2013	0.199 ***	0.040	2071.8	0.178 ***	0.032	1772.4	0.288	0.151	2795.3
λ	−0.321	0.197		0.136	0.122		−0.614	0.463	
θ1	0.361 ***	0.022		0.314 ***	0.013		0.276 ***	0.090	
θ2	3.056 ***	0.141		3.218 ***	0.086		2.042 ***	0.695	

The Extended Estimating Equations (EEE) model estimates are reported in the table; Adjusted Std. err. are standard errors adjusted for clustering at the patient level. *** *p* < 0.01, ** *p* < 0.05; UEBMI, Urban Employee-based Basic Medical Insurance scheme; URBMI, Urban Resident-based Basic Medical Insurance scheme; AD, Alzheimer’s disease; VaD, Vascular dementia; Others, other forms of dementia; Coef., Coefficient; Std. err., Standard errors.

**Table 6:** Factors associated with total inpatient costs including interaction terms (EEE Model).

Influential Factors	Overall		
	*n* = 5747		
	Coef.	Adjusted Std. err.	Marginal Effect
Gender			
Female (Reference)			
Male	0.057 **	0.026	532.7
Age group			
18 ≤ age < 50 (Reference)			
50 ≤ age < 60	0.022	0.103	209.1
60 ≤ age < 70	0.042	0.086	392.6
70 ≤ age < 80	0.055	0.081	514.6
80 ≤ age < 85	0.020	0.084	187.9
≥85	0.041	0.083	384.4
Insurance type			
URBMI (Reference)			
UEBMI	−0.013	0.051	−118.9
Disease type			
AD (Reference)			
VaD	0.133 ***	0.028	1283.8
Others	0.192 ***	0.031	1908.1
Comorbidities			
None (Reference)			
Hypertension	0.079 ***	0.026	737.1
Diabetes	0.054	0.036	516.9
Coronary	0.051	0.032	481.3
Hospital level			
Primary (Reference)			
Secondary	0.227 ***	0.034	2263.5
Tertiary	0.706 ***	0.049	6026.8
Length of stay (days)			
Days ≤ 10 (Reference)			
10 < Days ≤ 15	0.222 ***	0.059	2218.6
15 < Days ≤ 30	0.224 **	0.088	2183.4
30 < Days ≤ 60	−0.047	0.091	−428.0
>60 Days	0.313 ***	0.075	3294.8
Year			
year 2008 (Reference)			
year 2009	0.160 ***	0.040	1579.9
year 2010	0.148 ***	0.040	1470.8
year 2011	0.107 ***	0.037	1027.1
year 2012	0.137 ***	0.036	1318.0
year 2013	0.177 ***	0.036	1749.4
Insurance type × Length of stay (LOS)			
UEBMI × (10 < Days ≤ 15)	0.093	0.060	892.3
UEBMI × (15 < Days ≤ 30)	0.151	0.091	1452.4
UEBMI × (30 < Days ≤ 60)	0.347 ***	0.105	3775.4
UEBMI × (>60 Days)	0.824 ***	0.155	11,517.0
λ	−0.321	0.197	
θ1	0.361 ***	0.022	
θ2	3.056 ***	0.141	

The interaction terms between insurance type and length of stay were included in the Extended Estimating Equations (EEE) model; Adjusted Std. err. are standard errors adjusted for clustering at the patient level. *** *p* < 0.01, ** *p* < 0.05; UEBMI, Urban Employee-based Basic Medical Insurance scheme; URBMI, Urban Resident-based Basic Medical Insurance scheme; LOS, length of stay; AD, Alzheimer’s disease; VaD, Vascular dementia; Others, other forms of dementia; Coef., Coefficient; Std. err., Standard errors.

**Table 7:** Comparison of hospitalization costs for dementia patients by country.

Research	Country/Region	Year of Data	Costs in Article	Adjusted Costs *	Including Medication or Not
This Study	Guangzhou China	2013	CNY 9169.0	US$1479.8	YES
Hurd, et al. [13]	United States	2010	US$8946	US$9471.3	YES
Schwarzkopf, et al. [15]	Germany	2008	€ 3914	US$5403.9	NO
Kang, et al. [16]	Korea	2004	US$4296	US$5156.2	YES
Abdin, et al. [17]	Singapore	2013	US$7178	US$5521.5	YES
Gustavsson, et al. [18]	Northern Europe	2006	€ 651	US$905.9	NO
Gustavsson, et al. [18]	Western Europe	2006	€ 710	US$955.9	NO
Gustavsson, et al. [18]	Southern Europe	2006	€ 450	US$682.3	NO

Notes: Adjusted costs * were 2013 US dollar value using consumer price indices of study countries in the years of costs and purchasing power parity (PPP) exchange rate in 2013 from the Organization for Economic Co-operation and Development (OECD), based on the CCEMG-EPPI-Centre Cost Converter [26]. Northern Europe: Sweden, Denmark, UK, Belgium; Western Europe: Germany, Switzerland, the Netherlands, France; Southern Europe: Spain, Italy, Greece.
